# Diabetes Mellitus

**Published:** 1885-09

**Authors:** A. R. Davidson

**Affiliations:** Professor of Medical Chemistry and Toxicology, Niagara University; Physician to Buffalo Hospital of the Sisters of Charity


THE BUFFALO MEDICAL AND SURGICAL JOURNAL

Vol. XXVI. SEPTEMBER, 1885. No. 2.

### Original Communications.

### Diabetes Mellitus.

By A. R. Davidson, M. D.,*

Professor of Medical Chemistry and Toxicology, Niagara University; Physician to Buffalo Hospital of the Sisters of Charity.

The presence of an appreciable amount of sugar in the urine is the distinguishing characteristic of diabetes mellitus—usually there is also an increase in the amount of urine voided—but this "polyuria" is the chief distinctive feature of diabetes insipidus, and is the only important clinical fact common to the two affections.

The earliest reference to it is found in the works of Celsus, who speaks of an inordinate increase of urine leading to emaciation and endangering life. Galen and others of his time treat of the disease at greater length. Up to the middle of the seventeenth century, many and curious theories as to the cause and nature of the disease were held, the medical world having no suspicion that the urine contained sugar. The honor of this discovery belongs to an English physician, Thos. Willis. One hundred years after, Matthew Dobson, a physician practicing

* Read before the Buffalo Medical Club.

in Liverpool, discovered that the blood as well as the urine contained sugar in diabetes, and drew the conclusion that the sugar was not formed in, but only excreted by, the kidneys.

The first really important observation as to the treatment of the disease was made in 1776 by Dr. Rollo. a surgeon in the British army, who discovered that an animal diet not only reduced the quantity of urine voided, but also diminished the quantity of sugar daily eliminated, and he advanced the theory that vegetable food was converted into sugar by an abnormal gastric secretion. The immense stride taken by chemistry in the beginning of the present century, and its application to medicine, enabled the chemist to direct the investigations into an entirely new direction, and to correct the universally accepted theory that the sugar originated in the system from vegetable food only.

In 1848, Bernard made the grand discovery that animals as well as vegetables had a sugar-creating power; that the liver was daily and hourly as actively engaged in fabricating sugar as in secreting bile, and we now arrive at an entirely new phase in the literature of diabetes. Bernard's discoveries made this mysterious disease accessible to experiment, and pointed out the sources which would have to be investigated in the search for its origin.

A great store of facts as to the physiology of sugar formation and the pathology of the disease have been brought to light by subsequent investigations, but the final solution of the two great questions which are of the most importance to the pathology of the disease, are up to the present time still the subject of most eager research. The one *concerns the origin and purpose of glycogen in the system;* the other is in regard to the *nerve tracts which govern the distribution of the glycogen and sugar.*

Bernard proved conclusively that sugar is formed in the liver from a glycogenic or amyloid substance, and that this formation goes on not only after the death of the animal, but even after the removal of the liver from the body.

The question then arises, Is the accumulation of glycogen in the liver caused by the sugar which reaches the liver through the blood becoming directly re-converted into the starch-like glycogen and deposited in the hepatic cells, or do the amyloids taken as food prevent any demand upon the hepatic amyloid, which would otherwise be formed in the hepatic cells out of the breaking up of the protoplasm, thus supplying the needs of the economy?

Bernard found sugar to be present in the liver of dogs fed exclusively on animal diet, and was inclined to the opinion that the glycogen was unconnected with the amyloids taken as food. This erroneous idea led him into many difficulties while attempting to reconcile it with the well-known fact of the effects of different kinds of food upon diabetic patients.

Subsequent inquiries have established the following points:

When an animal is starved, the glycogen in the liver diminishes rapidly at first, more slowly afterwards. Even after some days of starvation, a little is frequently found, but in rabbits, at least, the whole may eventually disappear.

A diet of fibrine, and with fat and a little salt, does not appreciably increase the amount.

A meat diet does produce a small amount.

Cereals increase the amount considerably.

Sugar and starch, and especially sugar, cause a rapid and great augmentation in the quantity of glycogen in the liver.

We see, then, that when sugar is absorbed from the intestines, the quantity of amyloid in the liver is increased; and, from this fact, Dr. Pavy asserts that the liver, instead of allowing sugar to pass through it, and, also, producing sugar itself, really transforms the sugar which reaches it into amyloid substance; and that the blood of the hepatic vein, if care be taken to keep the animal in a perfectly normal condition, contains no more sugar than the blood of the portal vein.

Tscherinoff, McDonnell and others support this view; and, on the contrary, such recent writers as Harley, Thudicum and

Beale support the old opinion of Bernard, that the liver does, normally, discharge a certain quantity of sugar into the portal vein. I am disposed to adopt the theory that Dr. M. Foster advances, that both views are right.

A certain average composition of the blood is necessary, in order that the tissues may thrive upon it to the best advantage, and one element of that composition is a certain per cent. of sugar. We may suppose that the muscles, or other tissue, are continually drawing upon the blood for sugar, and the supply to meet the demand is chiefly supplied by the saccharine or starchy food taken into the system. When sugar is present in the blood beyond the needs of the economy, a portion is cast out by the kidney, while that passing by the portal vein is converted into glycogen; while a deficiency of the amount of sugar in the blood is at once supplied by the conversion of this hepatic glycogen.

Dr. Foster says "that this view that the glycogen of the liver is a reserve fund of carbo-hydrate material, is strongly supported by analogy, in the migration of starch in the vegetable kingdom. We know that the starch of the leaves of a plant, whether itself having passed through the glucose stage or not, is normally converted into sugar, and carried down to the roots, or other parts, where it frequently becomes once more changed back into starch; and when we consider that at every meal an excess of carbo-hydrate is thrown into the system, far more than is needed for the time being, and yet not more than will be needed before the next meal, it appears only natural that the excess should be at once, and without much labor, laid up as a store of carbo-hydrate, upon which the economy may make a call as occasion demands." This suggestion is supported by the fact that, whereas the glycogen in the liver varies much according to the food taken, that present in the muscles is fairly constant.

We may suppose, then, that the starch taken with the food is by the action of the salivary and pancreatic ferments hydrated into sugar, and the grape sugar thus carried to the liver by the portal vein is dehydrated into starch by the ferment action of the hepatic protoplasm.

Vegetable protoplasm can, undoubtedly, convert starch into sugar, and sugar into starch; and there is no reason to suppose that the animal protoplasm cannot also accomplish these changes.

A few words now as to the consideration of the second question: As to the nerve tracts which govern the distribution of the glycogen and sugar?

Experiments, to which I need not refer, have proved conclusively that the glycogenic function of the liver is subject to the influence of the nervous system, and particularly to a region of the cerebro-spinal centre, which is already known as the vaso-motor centre. How that influence is exerted, our want of knowledge is evidenced by the divergent opinions of the physiologists. Some consider it due to direct nervous action, and it has been supposed that a nerve centre, with afferent and efferent nerve fibres, preside over the actual process of secretion in some unexplained manner. According to Eckhard, the phenomena are those of irritation, and not of the simple withdrawal of any accustomed nervous influence. Beale, Cyr and Aladoff, on the contrary, regard the whole matter as simple loss of vascular tone, the diabetic puncture causing dilatation of the small branches of the hepatic artery. At present, we have no facts to show how, in diabetes, the excess of sugar in the blood arises. There seems to be ground for the opinion that there are two main varieties of the disease. One seems to be primarily due to disturbance of the functions of the liver, while the other can be traced to an original morbid condition of the brain. Clinical experience has shown that it *does* follow upon *nerve lesions* and irritation. Harley and other writers cite many cases following lesions of the fourth ventricle, tumors, clots, etc., in the brain. It is not unfrequently observed in cases of apoplexy. Conditions of the spinal marrow exercise but little influence, but the effect of certain nervous diseases is very decided. This has been shown, as to chorea, epilepsy, hysteria, delirium tremens, and acute mania, by Roberts, Harley Andral, Flint, and Cyr. Prout, Pavy and Dickinson rank mental disorders as amongst the most common causes.

Harley places the use of stimulants and disordered digestion as among the most frequent of the exciting causes; but Beale, M. H. Dickinson and Jules Cyr dispute this statement.

In some instances, diabetes seems traceable to hereditary constitutional peculiarity, and nearly all of the authors more or less strongly favor the hereditary nature of the disease.

We see, then, that diabetes springs from a multitude of causes: that the symptom of saccharine urine is not itself the disease, but merely the most prominent sign of the hidden complaint. It is, however, *the* diagnostic sign; and, as such, it is of the first importance to the practitioner to be able to certainly decide, not only the presence of sugar in the urine, but, also, in many cases, *the quantity*. And I will now show you a few of the many *tests for sugar* which have been proposed—and used. By far the most reliable and convenient, either for qualitative or quantitative estimation, is that modification of Trommer's, known as Fehling's. I will show you the *modus operandi* of that when I speak of quantitative examinations, but as it is not always on hand, I will give you, first, some of the simpler qualitative tests:

A very good one is Böttger's. This consists in adding, first of all, potash, then a small quantity of subnitrate of bismuth, and boiling the mixture; if sugar is present, we get a precipitate of suboxide of bismuth in the form of a black powder. Albumen will cause a black sulphide of bismuth to be formed. Ensure, therefore, the absence of albumen before employing the test.

That known as "Moore's" is a convenient rough test, but cannot be depended upon for detecting small quantities of sugar. It consists in adding to the urine suspected to contain sugar about half its bulk of liquor potassa; the mixture assumes a brown color upon boiling, if sugar is present.

As I before said, my own preference is decidedly for Fehling's test. This is composed of solutions of sulphate of copper, neutral tartrate of potash, and hydrate of soda. Its action depends upon the property which grape sugar has in the presence of a free alkali, and, at an elevated temperature, of depriving oxide of copper of one-half its oxygen, and thus converting it into red suboxide. When using the solution as a quantitative test, it is convenient to have it of such strength that ½ a grain of sugar shall exactly reduce the oxide contained in 100 grains of the solution, and to accomplish this, the following proportions are to be taken: Sulphate of copper, 94 grains; neutral tartrate of potassa, 378 grains; solution of caustic soda, sp. gr. 1.12, 4 ounces; add water to make 6 ounces fluid. If a small quantity of this be taken and heated to the boiling point in a test tube, the addition of a few drops of saccharine urine will cause the instant production of a brick red or copper color.

If the amount of sugar be small, this effect may not be apparent, and the liqu d should again be brought to the boiling point, and more urine added. The addition of a quantity of urine equal to the volume of test liquid employed, without the characteristic re-action, is certain evidence of the absence of sugar.

Let me here warn you against certain sources of error in performing this test.

1. The test solution, if kept for some time, may decompose, and sometimes may yield a deposit of sub-oxide when boiled alone. This is guarded against by keeping the solutions in separate bottles until required, and by always boiling the test fluid *first*, and adding thereto the urine. If before the addition of the urine there is any change in the blue color of the test upon boiling it, it should be rejected.

2. A small quantity of sugar will not be detected in an ammoniacal urine, and this would be a serious drawback to the test, were it not that sugar is very rarely found in such urine. Diabetic urine is almost always acid.

3. If albumen be present (a small amount will not prevent the detection of a considerable amount of sugar), the reduction of the copper does not take place. The albumen may, however, be readily removed by heat and acids and subsequent filtration, the free acid being neutralized by potash or soda, *but not by ammonia*, before the application of the test.

4. If the boiling is long continued, the copper may be reduced, when sugar is absent, by the action of other organic substances. Continued boiling is, however, entirely unnecessary in the application of the test, and should always be avoided.

5. Upon the addition of the alkaline test, there is always a precipitate of earthy phosphates, and the solution may change color upon boiling. This re-action, however, is only confusing to those inexperienced with the test. Occasionally, physicians bring me samples of urine which they believe to contain sugar, when it is quite free from it. They have used Fehling's test, and they say, "we get a precipitate and the blue color disappears." Remember that the re-action alone, characteristic of the presence of sugar, is the production of a brown or yellowish precipitate upon boiling the mixture not longer than a minute.

The application of this test to the estimation of the quantity of sugar present is very easy; all of the apparatus necessary is before you. This form of Burette (Bink's) I recommend to physicians, because it has its own stand,takes up little room, and is inexpensive. It is graduated to 250 grains. The urine having been diluted in the proportion of 1 to 10, the burette is filled up to 0. In this little flask, I place 200 grains of the Fehling's, prepared in accordance with the formula given above, and add to it an equal amount of pure water. The flask is then placed on a piece of wire gauze and gently heated to ebullition; allow now the fluid to flow from the burette into the boiling test solution; immediately the separation of the suboxide commences. The mixture appears reddish-brown, with a greenish tinge, and, upon the continued addition of the sugar solution, the precipitate assumes a deep red color. The moment this red color is apparent, the flask should be removed from the flame and the separated cupreous oxide allowed to settle. If the point of complete reduction has been nearly reached, the blue color of the supernatant solution will be observed only by holding the flask between the eye and the light. Again heat to ebullition, and add the urine drop by drop till the last blue shimmer disappears. The re-action is now finished. The 200 grains of test fluid used exactly correspond to 1 grain of sugar, and by reading off the number of grains added from the burette, the calculation may be easily made. Flint gives tables, in his little work on the chemical examination of urine, by which the little trouble of a calculation is avoided.

There is one more test to which I would call your attention, viz., the fermentation test. The application of this is very easy, and repeated experiments have proved its value. Into an 8-ounce vial, place 4 ounces of diabetic urine, and add to it a small lump of compressed yeast. The bottle is to be then closed with a nicked cork, to allow of the escape of gas, and placed in a warm place with a companion vial filled with the same urine, but tightly corked. After eighteen to twenty-four hours, the specific gravity of both is taken. The fermented specimen will be found to have lost weight, owing to the conversion of the sugar into alcohol and carbonic acid, and every degree lost, as expressed by the urinometer, will represent (very nearly) 1 grain of sugar per fluid ounce in the urine. Suppose, for example, that the specific gravity of the unfermented specimen is 1035, while that to which the yeast is added registers only 1015; the urine, therefore, contains 20 grains of sugar in each fluid ounce. Yeast can now always be had in the compressed form, and the only objection to the test is the time required to complete it.

While the recognition of sugar is the diagnostic sign of the disease, as a rule it is by other symptoms that the attention of the physician is directed to the urine, and yet, in many cases, these disturbances are so trifling as to give rise to no complaint at all, and the sugar is rather found accidentally. It should, therefore, be a rule with the physician, that in every case, when a complete examination of the patient is required, an examination of the urine should be made.

Usually the patient's attention is at first attracted to himself by an abnormal thirst and more frequent desire to pass urine. Not unfrequently he complains of a general lassitude, itching of the skin, and pains in the limbs, ascribed to rheumatism, and occasionally disturbances of vision first induce him to seek medical aid. Sometimes diabetes begins suddenly, but in general very gradually, and the disease in its beginning usually escapes observation. The extraordinary thirst and increase of the urinary secretion is often, for a long time, the only symptom which vexes the patient. As the disease progresses in severity, these symptoms increase, and the amount of urine voided is something enormous. Senator cites a case of a man, thirty-three years old, who passed 12 litres daily. Bence Jones saw it amount to 7 gallons. But these quantities are all belittled by Forsica's statement (I do not insist upon you believing it) that a diabetic voided 208 pounds of urine in a day.

As before stated, in some few cases the quantity of urine voided is normal, and it is very common to see a general subsidence of polyuria and the other diabetic phenomena some time before the fatal termination. Intercurrent febrile affections also usually mitigate all of the symptoms of the disease temporarily.

Emaciation is usually noticed quite early, although the appetite is increased not unfrequently to unappeasable hunger.

Nervous phenomena also appear — emotional derangement, severe neuralgia, and very commonly a decrease of the sexual instinct.

The skin becomes dry, harsh and rough; the patient seldom sweats, but on the contrary is much inclined to shiver and feel cold. The temperature of the body is very commonly below the normal 95 to 97.7. Very common accompaniments are digestive disturbances and derangements of the sense of sight, and finally signs of kidney affection, albuminous œdema and death. Usually, however, the patient does not die of the disease, but is carried off by some intercurrent affection in which the lungs or brain have become implicated.

In the majority of cases, however, the course of the disease, as thus outlined, may be greatly retarded and alleviated by judicious treatment; and in very many cases the disease may be completely arrested. The prognosis is extremely unfavorable, however, if the disease occurs in young persons. After forty, life may be indefinitely prolonged; but the younger the patient is, the more malignant and rapid is the course of the disease. In fat persons, the progress of the disease is usually slow, while lean persons yield more readily to it. A greater or less amount of sugar is not the most important fact. If due to the food ingested, a large amount is less significant than a much smaller proportion after the diet has been properly regulated. The quantity of urea eliminated is more important, as indicating the progress of disassimilation. If the azoturia is excessive, the prognosis is grave; but in estimating the amount of urea, due allowance must be made for the large increase due to a nitrogenous diet. A great deal depends upon the ability of the patient (both as to will power and fortune) to strictly follow advice as to the treatment of the disease; and this next demands our attention.

Like all intractable maladies, diabetes has had a great many remedies proposed for its treatment; each new one being introduced by some sanguine and incautious observer as a specific. But from what we now know of the pathology and history of the disease, we recognize that no one remedy, or particular line of treatment, is suitable to all cases of diabetes. Each individual case requires careful study, and our first effort must be, if possible, to detect and remove the cause of the accumulation of saccharine matter in the blood. If this cannot be accomplished, the ill effects may be, at least, mitigated by the use of sedatives. Pavy advocates the treatment with opium—but the use of it is open to serious objection. Codeia, in 1-grain doses, I have a partiality for, and it has been used in many cases with apparent benefit; it is free from many of the objections to opium. Tr. Cannabis Indica is commended by Harley; arsenite of bromine by Flint. Arsenic, itself, has some reputation, based upon the observation of Salkowsky, that glycogen diminishes in the liver of animals under the influence of arsenic. Creasotes, carbolic acid, sulphide of calcium, ergot and bromide of potassium are among the later remedies recommended on theoretical grounds, but experience has not demonstrated their value.

There are no remedies at present known that may be said to be curative of diabetes; but some remedies are useful. The employment of drugs must, however, be subordinate to what, in the present state of our knowledge, is by far the most important part of the treatment, viz., the dietetic treatment; and here success will depend upon the proper selection, preparation and combination of the food. That starchy and saccharine substances cause an increase in the quantity of sugar in the blood is proved beyond question, and every practitioner is familiar with the marked improvement in the condition of the diabetic patients when these and allied substances are withdrawn from his food. It requires, however, no little resolution on the part of the patient to persevere in this restricted diet, and the craving for bread, especially, becomes in many cases almost irresistible. The various bran flours are, most of them, as palatable as sawdust. The gluten flour of various manufacturers, all claiming to be free from starch, are absolutely unreliable, and often fraudulent; all those which I have examined contain large quantites of starch. If the patient will have bread, a small quantity of crust of bread, with plenty of butter, may be allowed under protest; but, as a rule, the craving for saccharine and starchy food gradually diminishes after a few weeks.

Aside from bread, the range of diet is large, and can be made endurable by an intelligent selection, on the part of the physician, from the long list of articles of food which contain no starch or sugar, or in so small a quantity as to be practically unobjectionable. I have frequently seen patients who complained that they were being starved, and that they would rather die than continue the diet ordered by their physician; and usually in such cases they have been restricted to the exceedingly limited diet of meat, eggs and fats. That such a rigorous diet is unnecessary, the following bill of fare, compiled by Dr. Flint from the tables given by Prof. Bouchardat, of Paris, and other authorities, is sufficient proof:

### Diet-Table.

Breakfast.—Oysters stewed, without milk or flour; clams stewed, without milk or flour.

Beefsteak, beefsteak with fried onions, broiled chicken, mutton or lamb chops, kidneys (broiled, stewed or deviled), tripe, pig's feet, game, ham, bacon, deviled turkey or chicken, sausage, corned-beef hash without potato, minced beef, turkey, chicken or game with poached eggs.

All kinds of fish, fish-roe, fish-balls without potato.

Eggs cooked in any way except with flour or sugar, scrambled eggs with chipped smoked beef, picked salt cod-fish with eggs, omelets plain or with ham, with smoked beef, kidneys, asparagus-points, fine herbs, parsley, truffles or mushrooms.

Radishes, cucumbers, water-cresses, butter, pot-cheese.

Tea or coffee, with a little cream and no sugar. (Glycerine may be used instead of sugar, if desired.)

Light red wine for those who are in the habit of taking wine at breakfast.

Lunch or Tea.—Oysters or clams cooked in any way except with flour and milk, chicken, lobster or any kind of salad except potato, fish of all kinds, chops, steaks, ham, tongue, eggs, crabs or any kind of meat, head-cheese.

Red wine, dry sherry or Bass's ale.

Dinner.—Raw oysters, raw clams.

*Soups.*—Consomme of beef, of veal, of chicken or of turtle, consomme with asparagus-points, consomme with okra, ox-tail, turtle, terrapin, oyster or clam without flour or milk, chowder without milk or potato, mock turtle, mullagatawny, tomato, gumbo fillet.

*Fish, etc.*—All kinds of fish, lobsters, oysters, clams, terrapin, shrimps, craw-fish, hard-shell crabs, soft-shell crabs. (No sauces containing flour or milk.)

*Relishes.*—Pickles, radishes, celery, sardines, anchovies, olives.

*Meats.*—All kinds of meat cooked in any way except with flour, all kinds of poultry without dressings containing bread or flour, calf's-head, kidneys, sweet-breads, lamb-fries, ham, tongue, all kinds of game, veal, fowl, sweet-breads, etc., with currie, but not thickened with flour. (*No liver.*)

*Vegetables.*—Truffles, lettuce, romaine, chiccory, endive, cucumbers, spinach, sorrel, beet-tops, cauliflower, cabbage, Brussels-sprouts, dandelions, tomatoes, radishes, oyster-plant, celery, onions, string-beans, water-cresses, asparagus, artichauts, Jerusalem artichokes, parsley, mushrooms, all kinds of herbs.

*Substitutes for Sweets.*—Peaches preserved in brandy without sugar, wine-jelly without sugar, gelee au kirsch without sugar, omelette au rhum without sugar, omelette a la vanille without sugar, gelee au rhum without sugar, gelee au cafe without sugar.

*Miscellaneous.*—Butter, cheese of all kinds, eggs cooked in all ways except with flour or sugar, sauces without sugar, milk or flour.

Almonds, hazel-nuts, walnuts, cocoanuts.

Tea or coffee with a little cream and without sugar. (Glycerine may be used instead of sugar, if desired.)

Moderately palatable ice-creams and wine-jellies may be made, sweetened with pure glycerine; but although these may be quite satisfactory for a time, they soon become distasteful.

*Alcoholic Beverages.*—Claret, burgundy, dry sherry, Bass's ale or bitter beer. (No sweet wines.)

PROHIBITED.

Ordinary bread, cake, etc., made with flour, sugar; desserts made with flour or sugar; vegetables, except those mentioned above; sweet fruits.

When under dietetic treatment, the quantity of urine becomes normal and the sugar has ceased to be eliminated, some food containing starch may be used cautiously, the urine being examined three or four hours after eating, in order to determine the effect upon the diabetic condition. It is well to allow periods of moderately restricted diet to alternate with the vigorous diet.

During the periods when greater liberty is allowed, or when the restricted diet fails to control the disease, some of the various drugs may be advantageously employed—codeia or opium, quinine, chalybeates, nerve tonics, etc., according to the various indications for medical treatment. Bodily exercise, particularly of the arms, is to be advised. Measures to maintain the warmth and functions of the skin are, at all times, specially important. Warm baths are very serviceable, and warm clothing and avoidance of cold is to be insisted upon, in view of the tendency toward pulmonary trouble.

